# A Ratiometric Fluorescence Probe for Selective Detection of ex vivo Methylglyoxal in Diabetic Mice

**DOI:** 10.1002/open.202200055

**Published:** 2022-05-11

**Authors:** Qunfang Xie, Yuanjin Zhan, Longhua Guo, Huili Hao, Xianai Shi, Jianmin Yang, Fang Luo, Bin Qiu, Zhenyu Lin

**Affiliations:** ^1^ Department of Cadre's Ward The First Affiliated Hospital of Fujian Medical University Fuzhou Fujian, 350005 P. R. China; ^2^ Institute of Nanomedicine and Nanobiosensing; MOE Key Laboratory for Analytical Science of Food Safety and Biology Fujian Provincial Key Laboratory of Analysis and Detection Technology for Food Safety College of Chemistry Fuzhou University Fuzhou 350116 P. R. China; ^3^ College of Biological, Chemical Sciences and Engineering Jiaxing University Jiaxing Zhejiang 314001 P. R. China; ^4^ College of Biological Science and Engineering Fuzhou University Fuzhou Fujian 350116 P. R. China

**Keywords:** diabetes, 2,3-diaminonaphthalene, imaging, methylglyoxal, ratiometric fluorescence

## Abstract

Accurate monitoring of methylglyoxal (MGO) at cell and living level was crucial to reveal its role in the pathogenesis of diabetes since MGO was closely related to diabetes. Herein, a ratiometric fluorescence strategy was constructed based on the capture probe 2,3‐diaminonaphthalene (DAN) for the specific detection of MGO. Compared to the fluorescent probes with a single emission wavelength, the ratiometric mode by monitoring two emissions can effectively avoid the interference from the biological background, and provided additional self‐calibration ability, which can realize accurate detection of MGO. The proposed method showed a good linear relationship in the range of 0–75 μm for MGO detection, and the limit of detection was 0.33 μm. DAN responded to MGO with good specificity and was successfully applied for detecting the ex vivo MGO level in plasma of KK−Ay mice as a type II diabetes model. Besides, the prepared DAN test strip can be visualized for rapid semi‐quantitative analysis of MGO using the naked eye. Furthermore, human skin fibroblasts and HeLa cells were utilized for exogenous MGO imaging, and ex vivo MGO imaging was performed on tissues of KK−Ay mice. All results indicated that the DAN‐based ratiometric fluorescence probe can be used as a potential method to detect the level of MGO, thus enabling indications for the occurrence of diabetes and its complications.

## Graphical abstract

**A ratiometric fluorescence probe** with a large Stokes shift for specific detection of diabetes‐related methylglyoxal was obtained. 2,3‐Diaminonaphthalene exhibited highly sensitive and anti‐interference ability for methylglyoxal detection by naked‐eye readout. This probe was thus capable of exogenous imaging methylglyoxal in living cells and ex vivo imaging methylglyoxal in the tissues of type II diabetes model mice.

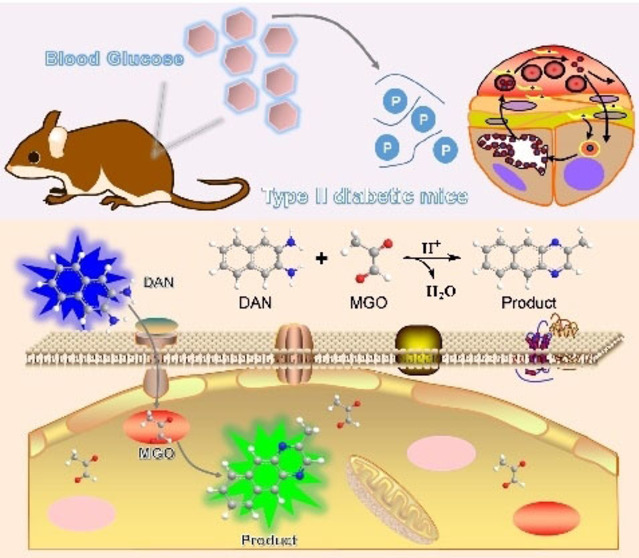

## Introduction

Methylglyoxal (MGO) is the active dicarbonyl in the metabolism of glucose, fatty acids, and amino acids in all living cells and the main precursor of advanced glycation end products (AGEs).[^1^, ^2^] MGO is closely related to diabetes, and MGO levels in the plasma of diabetics patients were found to change at different stages.^[3]^ Besides, MGO can induce complications related to diabetes, such as cardiovascular disease (CVD),^[4]^ Alzheimer's disease (AD),^[5]^ age‐related diseases,[^6^, ^7^] and has a significant impact on the cellular system.[^8^, ^9^, ^10^] So far, there is still a lack of clear understanding of the pathogenesis and interaction pathways associated with MGO, mainly because of the lack of effective methods for monitoring MGO content in different stages of the complex systems.[^11^, ^12^, ^13^] Therefore, the accurate and reliable determination of MGO content is of great value in exploring its physiological significance and developing strategies for preventing diabetes and complications.

Previously reported methods for determining MGO levels include chromatography,[^14^, ^15^, ^16^] electrochemistry,^[17]^ fluorescent probes,[^18^, ^19^] and colorimetry.^[20]^ Yet, most of the methods are only suitable for the evaluation of in vitro serum samples, which limits the understanding of its pathological and physiological significance at the cellular and living level. As an irreplaceable and indispensable tool, fluorescent probe imaging technology can intuitively and conveniently be used to perform real‐time dynamic three‐dimensional observation and monitoring of intracellular systems and in vivo biologically active compounds or biological functional guest components, with the advantages of real‐time visualization, good specificity, non‐invasiveness, and high spatial and temporal resolution.[^21^, ^22^, ^23^] Spiegel et al. reported the first visual and turn‐on fluorescent sensor for monitoring and imaging of MGO levels in live cells.^[24]^ It opened up a new strategy to reveal the potential association between MGO and diabetes from the biological system level. However, the fluorescent probes with a single emission wavelength were susceptible to severe interference from the biological tissue absorption and tissue autofluorescence interference in the microenvironment of cells and biological systems, thereby affecting the accuracy of the detection results.[^25^, ^26^] The ratiometric fluorescent probes developed in the past decade have gradually attracted extensive research interest.

**Scheme 1:**
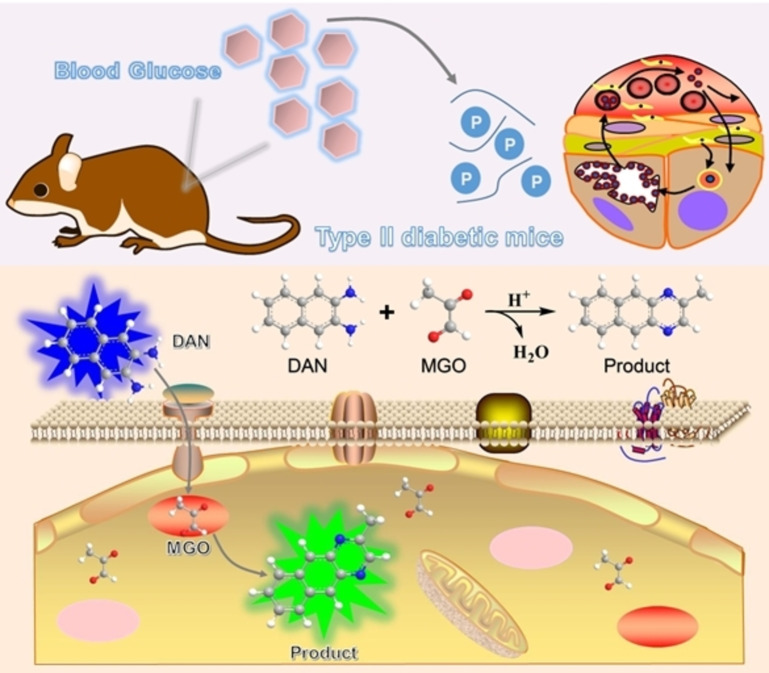
Selective in vivo monitoring of ex vivo MGO in diabetic mice by a DAN‐based ratiometric imaging.

Compared with single‐photon fluorescent probes, the ratiometric mode by monitoring two (or more) emissions provided built‐in self‐calibration for signal correction, enabling more sensitive and reliable detection, which has been widely used in clinical diagnosis, sensing, and imaging.[^27^, ^28^, ^29^, ^30^] Herein, 2,3‐diaminonaphthalene (DAN) with an effective highly conjugated structure and long emission wavelength was designed as the ratiometric fluorescent capture probe for responding to MGO. As the strategy proposed in Scheme 1, a two‐step Schiff's base reaction with MGO under physiological pH condition was used to form 2‐methylbenzo[g]quinoxaline. The conjugation degree of the adduct was further increased, and the emission wavelength underwent a significant redshift. Meanwhile, the fluorescence intensity from DAN gradually decreased, and the fluorescence intensity of the adduct gradually increased, the ratiometric measurements can achieve more accurate quantitative tracking of MGO in biological samples. Afterward, an MGO test strip was prepared to realize the visual analysis of MGO content in serum samples, which was fast, easy to operate, and low in cost, and can further facilitate MGO level evaluation. Furthermore, the proposed method had been applied to detect and image the intracellular exogenous MGO in human skin fibroblasts (HSF) and HeLa cells. Finally, the proposed method was applied for assessing the content of MGO in the serum and to perform ratiometric imaging of ex vivo MGO in various tissues of type II diabetic mice model. It can be seen that the ratiometric fluorescent probe can be used for the measurement and imaging of MGO levels in cells and tissues, providing potential help for exploring the development mechanism of MGO‐related diabetes.

## Results and Discussion

### Feasibility of MGO Detection Based on the Ratiometric Fluorescent Probe DAN

**Figure 1:**
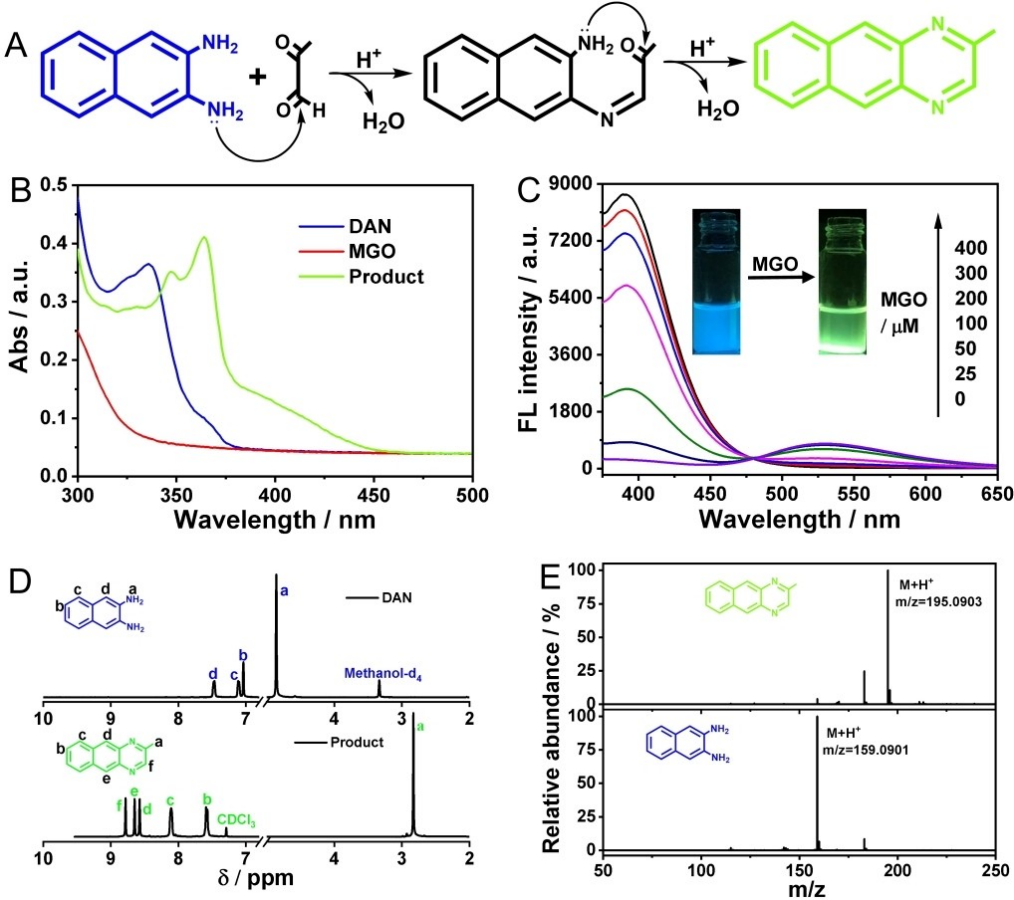
Feasibility of the proposed fluorescence probe. (A) Reaction mechanism of DAN towards MGO. UV‐Vis spectra (B) and FL spectra under 365 nm excitation (C) in response to different concentrations of MGO. ^1^H NMR and high‐resolution mass spectra of DAN and product. (D) Partial ^1^H NMR spectra (400 MHz). (E) HR‐MS spectra.

As shown in Figure 1B, after the interaction between DAN and MGO, the characteristic absorption peaks are significantly red‐shifted. Meantime, the fluorescence intensity of DAN (at 390 nm) gradually decreased, while the adduct (at 530 nm) gradually increased, and the fluorescence color changed from blue to green (Figure 1C). Figure S1 (Supporting Information) shows the fluorescence properties of DAN and the adduct. The addition product was separated and purified with a silica gel chromatography column and used for further characterization. As presented in the ^1^H NMR spectrum in Figure 1D, after the reaction with MGO, the NH_2_ proton resonance peak at 4.86 ppm in DAN disappeared, and a new CH_3_ proton resonance peak at 2.83 ppm was generated in the product. Moreover, the aromatic proton signal in DAN shifted downfield in the spectrum of the product. Then the mass‐to‐charge ratio (m/z) of DAN and product were analyzed by using high‐resolution mass spectrometry (HR‐MS). As shown in Figure 1E, the product showed a strong peak at m/z of 195.0903, compared with the characteristic peak of DAN (m/z=159.0901), also indicating that reaction between DAN and MGO had led to loss of two water molecules and formed quinoxaline (Figure 1A). Also, ^13^C NMR spectroscopy was used to gain further evidence for the assignment of green phosphor as the product of the reaction (Figure S2). The above results confirmed that the ratiometric fluorescence probe was feasible and the sensing mechanism was as described in Scheme 1.

### Performance of the Ratiometric Strategy for MGO Detection

**Figure 2:**
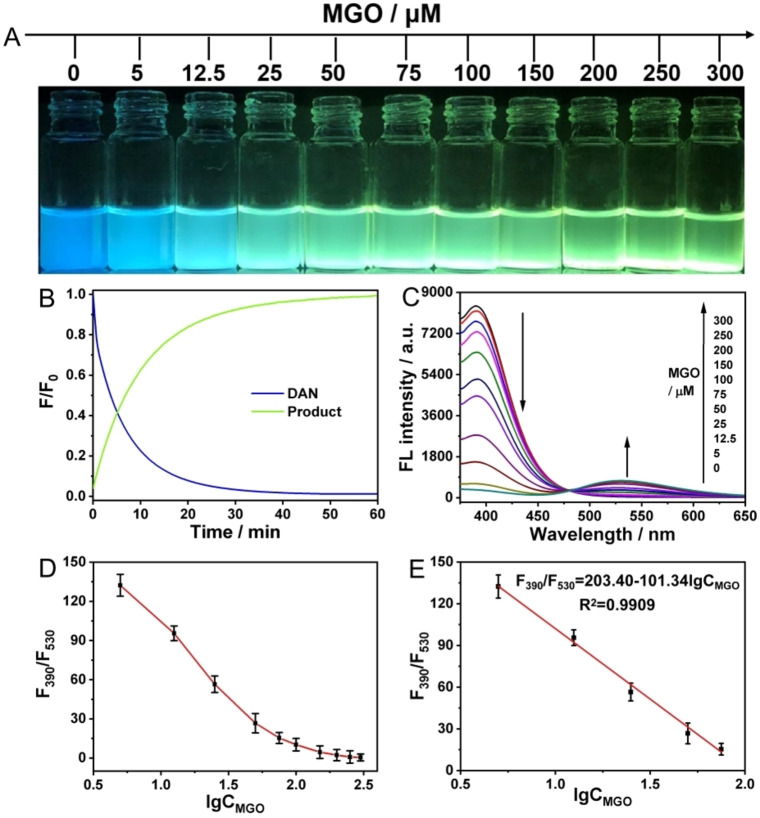
Performance of the ratiometric fluorescent probe DAN in response to MGO in PBS buffer (10 m **m**, pH 7.4, containing 0.05 % DMSO) at 37 °C. (A) The image showed the color change of the solution after the reaction of different concentrations of MGO (from left to right: 0, 5, 12.5, 25, 50, 75, 100, 150, 200, 250, and 300 μm, respectively). (B) The dynamic curve of fluorescence intensity with reaction time. (C) Fluorescence spectrum under 365 nm excitation of DAN (100 μm) in response to different concentrations of MGO (0–300 μm), respectively. (D) and (E) Plot of fluorescence intensity toward MGO concentrations.

It is well known that detection efficiency is critical in practical applications. As can be seen in Figure 2B, DAN reacted with excess MGO and the fluorescence intensity of DAN decreased rapidly by about 100 % within 60 min, while the fluorescence intensity of the product was significantly enhanced, indicating that DAN reacted with MGO quickly and efficiently. As shown in Figure 2C, as the MGO content increases, the intensity of the fluorescence of DAN gradually decreased, and the corresponding intensity of the product gradually increased, while the fluorescence color changed progressively from blue to green (Figure 2A). The ratiometric fluorescence intensity F_390_/F_530_ was negatively correlated with the MGO log concentration in the range of 0–75 μm (R^2^=0.991, Figures 2D and 2E), and the limit of detection (LOD) was as low as 0.33 μm (3σ/κ). At the same time, the changing trend of UV‐Vis absorbance value with MGO concentration was consistent with fluorescence intensity (Figure S3). Furthermore, the traditional OPD method was additionally used for MGO evaluation. It is worth noting that the characteristic absorption peaks of the adduct between OPD and MGO were all in the ultraviolet region, and the real serum sample and intracellular matrix environment will cause great interference and significantly affect the accuracy (Figure S4). In contrast, the ratiometric strategy proposed in this work had self‐correcting capabilities on the one hand. On the other hand, the characteristic emission peak of the adduct of DAN and MGO was located in the long‐wavelength region (530 nm), which can significantly avoid the interference of serum and intracellular medium. As listed in Table S1 (Supporting Information), the linear range and LOD of the proposed method are comparable to those of the previously reported detection methods and even have better‐sensing performance. Besides, previous studies found that higher MGO levels were observed in the plasma of patients with diabetes, and the plasma concentration was higher than 600 nm, indicating that this method is fully capable of testing requirements. Moreover, the entire detection procedure can be completed within 1 h, and the complex electrode modification^[17]^ and nanomaterial preparation process^[20]^ are not required, which can greatly improve the efficiency of actual detection.

### Specificity and Selectivity of DAN toward MGO

**Figure 3:**
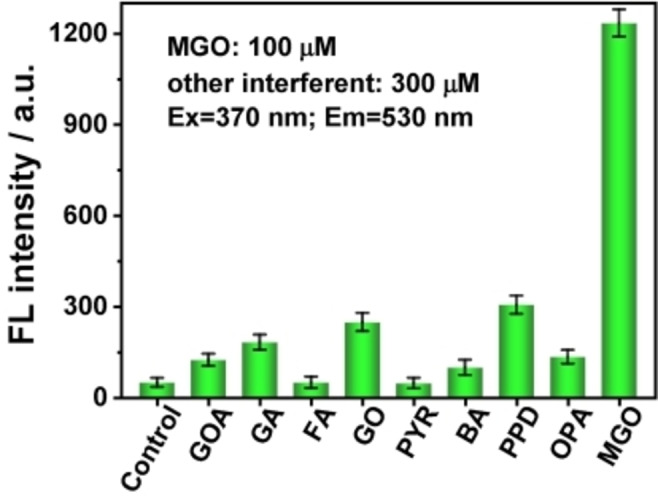
Specificity and selectivity of DAN in response to MGO (100 μ **m**) and various active aldehydes (300 μm) including glyoxylic acid (GOA), glutaraldehyde (GA), formaldehyde (FA), glyoxal (GO), pyruvate (PYR), benzaldehyde (BA), 1‐phenyl‐1,2‐propanedione (PPD), and *o*‐phthalaldehyde (OPA), respectively.

The above results indicated that the DAN‐based probe exhibited excellent sensing performance for MGO under ideal solution conditions. Given the composition of the real biological sample and the complexity of the microenvironment in vivo, a series of substances with active carbonyl were investigated to verify the specificity of DAN under the same conditions. At the same time, some ions and amino acids commonly found in serum were chosen to evaluate the anti‐interference ability of DAN in response to MGO. As shown in Figure 3, the fluorescence intensity change value of the adduct between DAN and MGO was significantly higher than that of other aldehydes, suggesting that DAN had high specificity for MGO recognition.

OPD is a common reactive group that responded to nitric oxide (NO). The p*K*_a_ of adduct‐triazole is approximately 7.7, and its deprotonation at physiological pH (pH 7.4) will lead to the formation of electron‐rich triazole salts.^[24]^ The electron‐rich system induced fluorescence quenching through the photoinduced electron transfer (PET) mechanism.^[31]^ On the other hand, due to the lack of acidic protons in the adduct quinoxaline of DAN and MGO, it will not undergo a quenching process similar to NO. Therefore, under physiological conditions (pH 7.4), the fluorescence response of DAN to MGO was much stronger than that of NO (Figure S5). Moreover, a large number of studies have found that the ability of vascular endothelial cells to synthesize NO in diabetic rats is reduced, and the NO synthesized by vascular endothelium is only 15 % of the normal content, or even lower.^[32]^ In addition, oxygen free radicals (ROS)^[33]^ and glycation end products^[34]^ will also reduce the content of NO during the development of diabetes. The MGO content of diabetic patients was at least two orders of magnitude higher than the physiological concentration of NO.^[35]^ In conclusion, low NO levels in diabetic patients did not significantly affect the two‐photon ratiometric detection of MGO. The interference from common metal ions, anions, amino acid, reactive oxygen species (ROS), and their mixed solutions on DAN recognition of MGO was found to be almost negligible (Figure S6). Hence, these results indicated that NO will not interfere with the detection of MGO by DAN. These results strongly confirmed the specificity and anti‐interference ability of DAN in response to MGO, which can be used for the analysis of diseases related to MGO.

### Determination of ex vivo MGO Contents in the Serum of Type II Diabetic Mice

KK−Ay mice were chosen as a model of type II diabetes[^36^, ^37^, ^38^, ^39^] and the ex vivo MGO content in plasma was determined. After centrifugation of the plasma, the serum of four mice was repeatedly measured three times, and the test results were listed in Table 1. The recovery rates were in the range of 96.80–106.20 % and the RSD (n=3) was lower than 6.7 %. Moreover, the MGO content in the same plasma samples was additionally evaluated by the mice MGO ELISA kit (Figure S7). The measurement results were not significantly different from the proposed method, indicating the authenticity of the MGO content, and it was consistent with the reported results.^[40]^ Therefore, the DAN‐based ratiometric probe can be considered as one of the potential methods for determining MGO in biological samples.

**Table 1:** The ex vivo MGO concentrations in type II diabetic mice plasma estimated by DAN and ELISA method.

KK−Ay mice No.	Detected/μm	Spiked/μm	Total found/μm	Recovery/%	RSD/%	Mice MGO ELISA kit^[a]^/μm
**1**	0.696	1.0	1.672	97.60	5.2	1.593
		2.0	2.763	103.35	3.8	2.824
		5.0	5.756	101.20	6.7	5.814
**2**	0.823	1.0	1.858	103.50	2.4	1.735
		2.0	2.759	96.80	4.3	2.783
		5.0	5.739	98.32	5.6	5.966
**3**	0.518	1.0	1.580	106.20	3.7	1.475
		2.0	2.589	103.55	6.2	2.641
		5.0	5.623	102.10	5.1	5.598
**4**	0.428	1.0	1.411	98.30	6.3	1.513
		2.0	2.521	104.65	4.4	2.492
		5.0	5.554	102.52	5.7	5.508

^[a]^ Each serum sample was tested at an appropriate dilution and measurements were conducted in triplicate.

### Multicolor Fluorescent Test Strips for Visual Analysis of MGO

**Figure 4:**
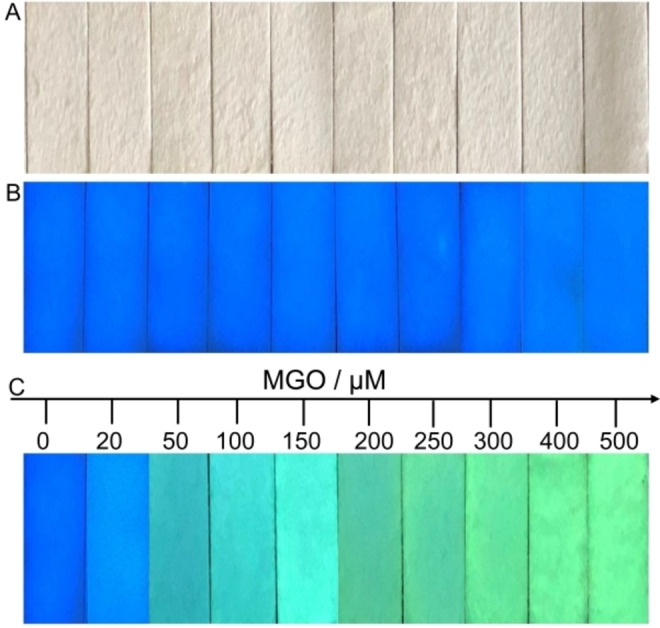
Multicolor fluorescent test strips for visual analysis of MGO. Pictures of DAN based test strips under visible light (A) and 365 nm UV light (B). (C) Photos of the ratiometric test strips under 365 nm UV light in response to MGO in the range of 0–500 μm.

To simplify the measurement procedure to meet the requirements of rapid and convenient analysis with the naked eye in‐house and in specific situations, we aimed at constructing a portable detection device for MGO level assessment. Given the low cost, non‐toxicity, no background fluorescence, large adsorption load capacity and possibility to be cut to any size and shape of filter paper, the fluorescence capture probe DAN was successfully immobilized by soaking and drying on a filter paper as a carrier (Figure 4A). As displayed in Figure 4B, the blue fluorescence of the test strip loaded with DAN was very uniform. When the different concentrations of MGO solution were added and reacted for 1 h, the fluorescent color of the test strips gradually changed from blue to green (Figure 4C). Not only can semi‐quantitative analysis of visualization be achieved, it can also meet the requirement of lack of equipment and on‐site rapid detection. Due to the good portability of the DAN‐based test strips and color‐dependent concentration that can be easily distinguished by the naked eye, this is of great significance and potential for the visual evaluation of MGO content.

### Ratiometric Imaging of MGO

**Figure 5:**
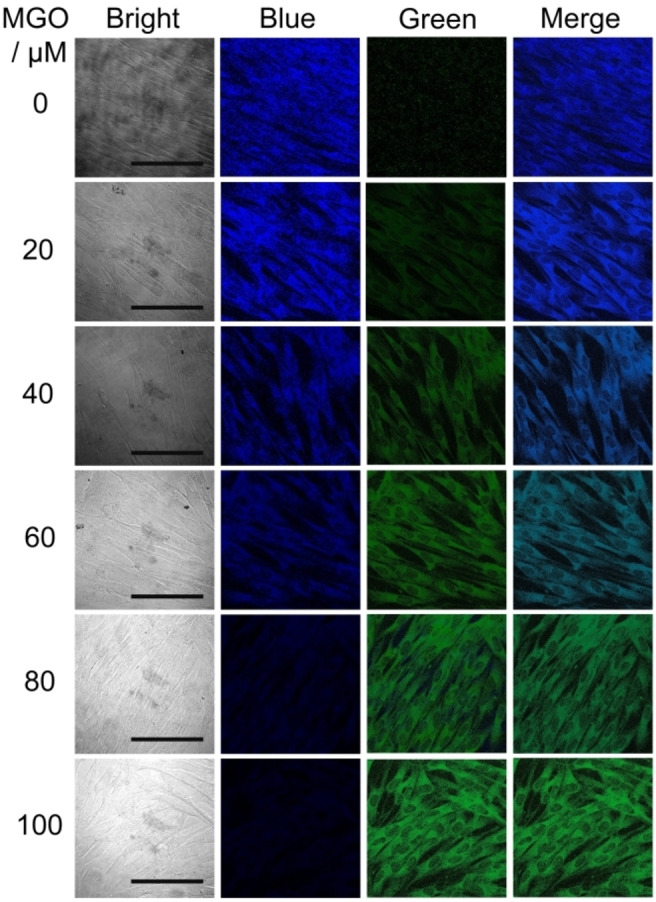
Ratiometric imaging of exogenous MGO in HSF cells. The excitation and emission wavelength of the blue channel (DAPI) were located at 359 nm and 461 nm, respectively. The excitation and emission wavelength of the green channel (FITC) were located at 496 nm and 518 nm, respectively. Scale bar: 50 μm.

The close relationship between MGO and diabetes required visual observation from the cellular level, tissue level and in vivo to explain its pathological and physiological significance. Prior to exogenous MGO imaging, it is first necessary to investigate the cytotoxicity of exogenous ingested DAN and MGO by MTT assay. In this work, HSF and HeLa cells were selected for intracellular fluorescence imaging. Figure S8 revealed that the cell viability of HSF cells after being incubated with 0–100 μm DAN and MGO for 24 h was still greater than 90 %. The cytotoxicity of DAN and MGO against HeLa cells were further investigated under the same conditions. The results of MTT assay were similar to those of HSF cells (Figure S9). Moreover, cell death and death staining results HSF cells were incubated with 100 μm DAN solution for 6 h and rinsed with PBS, and further incubated with different concentrations of MGO for 6 h. Finally, after PBS cleaning, blue fluorescence and green fluorescence changes were observed by fluorescence confocal microscopy. Figure 5 further demonstrates the low toxicity of DAN and MGO (Figures S10 and S11) and that DAN can be applied for intracellular imaging the fluorescence intensity of the blue and green channels, showing a trend of decreasing and increasing respectively as the MGO concentration gradually increases. At the same time, HeLa cells were also examined for confocal imaging of exogenous MGO, and the imaging results were similar to those of HSF cells (Figure S12). The above‐detailed results indicate that DAN and MGO are membrane‐permeable and specifically interacted within the cell. From the perspective of the overlay channel, the entire process of recognizing MGO was that the fluorescent color changed from blue‐cyan‐green.

**Figure 6:**
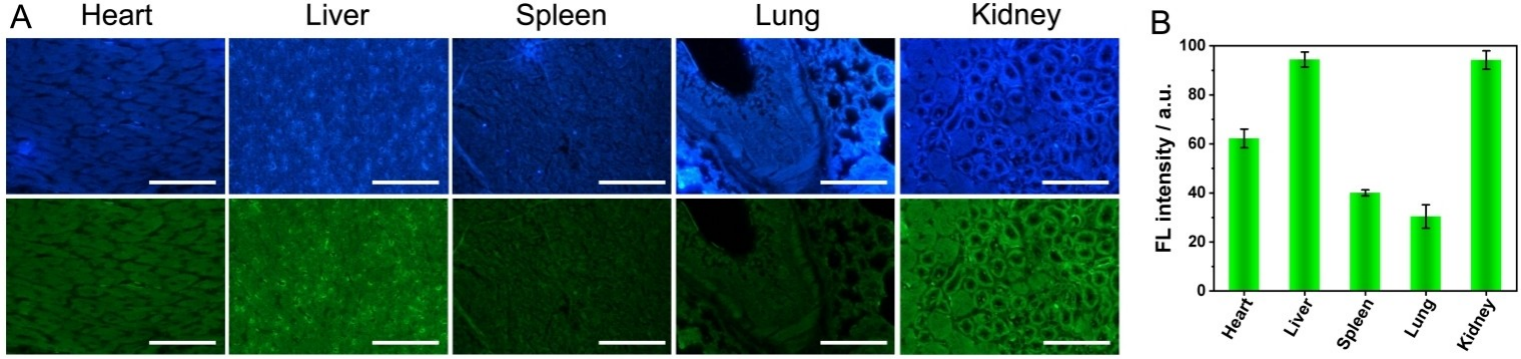
(A) Ratiometric fluorescence imaging of MGO level in type II diabetic mice tissues (heart, liver, spleen, lung, and kidney). The excitation and emission wavelength of the blue channel (DAPI) were located at 359 nm and 461 nm, respectively. The excitation and emission wavelength of the green channel (FITC) were located at 496 nm and 518 nm, respectively. (B) Fluorescence intensity of MGO adducts in different tissues. Scale bar: 50 μm.

Disordered glucose metabolism and its complications cause abnormal MGO levels. KK−Ay mice were used as a model of type II diabetes, focusing on changes in MGO levels in tissues induced by diabetes. The heart, liver, spleen, lungs, and kidneys of KK−Ay mice were extracted and sectioned. Tissue imaging was performed after incubation with 100 μm DAN solution at 37 °C for 1 h. As can be seen from Figure 6, the MGO content in the liver, kidney, and heart of KK−Ay mice was relatively high, while the content of lung and spleen was relatively low, which was consistent with the distribution of MGO content in different tissues reported in the previous literature.^[18]^ This was mainly the tissue in which the liver and kidney were the main metabolic and excretory functions, and the heart was mainly the tissue of metabolism and circulatory function, which was beneficial to the accumulation of a large amount of MGO.[^41^, ^42^, ^43^] It was worth noting that, differing from the reported literature, a relatively high MGO content was observed in the mice heart. This was because the ratiometric fluorescent probe can be used to image and analyze the MGO content in the tissue more clearly and accurately. The above results demonstrated that DAN‐based ratiometric fluorescent probes can be applied as a potentially effective tool for the assessment of exogenous and ex vivo MGO levels. Moreover, MGO imaging in ex vivo tissues provided a potential means to further reveal the pathogenesis of diabetes and its complications.

## Conclusion

In this work, a ratiometric fluorescence approach was developed for the specific sensing of MGO based on the interaction between DAN and MGO. In the presence of MGO, the conjugation degree of the product was increased, and the fluorescence emission wavelength was red‐shifted up to 140 nm, accompanied by a change in the ratio of the fluorescence intensity. Moreover, DAN had good specificity of recognition of MGO and excellent anti‐interference ability, and it has been successfully used for the detection of MGO content in the serum of KK−Ay mice as a type II diabetes model. Additionally, the prepared DAN test strip can realize visualized rapid semi‐quantitative analysis by the naked eye, and it can meet the requirements of only very little needed equipment for rapid on‐site detection. To further visualize and reveal the pathological and physiological significance of diabetes and its complications, HSF and HeLa cells were chosen for exogenous MGO imaging, and MGO imaging of ex vivo tissues of KK−Ay mice such as heart, liver, spleen, lung, and kidney. The results showed that there was a large accumulation of MGO in the heart, liver, and kidney of KK−Ay mice. All these results indicated that the DAN‐based ratiometric fluorescent probe can be used as a potentially effective tool for the assessment of MGO levels, and maybe a potential means for further investigation of the causes and development mechanism of diabetes and its complications.

## Experimental Section

### Determination of Exogenous MGO

DAN (1.0 mm, 20 μL) and different concentrations of MGO (0, 5, 12.5, 25, 50,75,100, 150, 200, 250, and 300 μm) were sequentially added to PBS buffer (10 mm, pH 7.4, containing 0.05 % DMSO). The mixed solution was vortexed and reacted at 37 °C for 1 h, and the changes in fluorescence intensity of the mixed solution were measured and scanned.

### Ratiometric fluorescence confocal imaging of exogenous MGO level in HSF and HeLa cells

Cell suspension (HSF and HeLa) were cultured in 24‐well plates at the same cell concentration and operating conditions. After the cells grew to 80 %, the culture solution was discarded and replaced with 100 μm DAN (diluted in the medium), and cultured at 37 °C for 6 h. Then, removed the DAN solution and added 1.0 mL of different concentrations of MGO solution (0, 20, 40, 60, 80, and 100 μm, diluted in the medium), and continued to culture at 37 °C for 6 h. Finally, after PBS cleaning, blue fluorescence and green fluorescence changes were observed by laser confocal fluorescence microscopy.

### Fluorescence Confocal Imaging of ex vivo MGO Level in Type II Diabetic Mice

The KK−Ay mice were selected as a type II diabetes model, the mice were dissected and the heart, liver, spleen, lungs, and kidneys were taken for tissue sectioning. The prepared tissue sections were individually placed in glass Petri dishes containing DAN (100 μm, 15 mL) PBS buffer solution, and then incubate at 37 °C for 1 h. After removal, the tissue sections were rinsed with PBS, and finally, a blue and green ratiometric channel were recorded using a fluorescence inverted microscope.

## Conflict of interest

The authors declare no conflict of interest.

1

## Supporting information

As a service to our authors and readers, this journal provides supporting information supplied by the authors. Such materials are peer reviewed and may be re‐organized for online delivery, but are not copy‐edited or typeset. Technical support issues arising from supporting information (other than missing files) should be addressed to the authors.

Supporting Information

## Data Availability

The data that support the findings of this study are available in the supplementary material of this article.
