# Supplementary material for: A Ratiometric Fluorescence Probe for Selective Detection of ex vivo Methylglyoxal in Diabetic Mice
Source: ChemistryOpen. 2022 May 11;11(5):e202200055. doi: 10.1002/open.202200055 (PMC9092288; doi:10.1002/open.202200055)
Supplement: Supplementary file 1 — Supporting Information [file OPEN-11-e202200055-s001.pdf]

ChemistryOpen

Supporting Information

A Ratiometric Fluorescence Probe for Selective Detection of ex vivo Methylglyoxal in Diabetic Mice

Qunfang Xie[+], Yuanjin Zhan[+], Longhua Guo,* Huili Hao, Xianai Shi, Jianmin Yang,* Fang Luo, Bin Qiu, and Zhenyu Lin*

Table of contents

S1 **Table of contents**

S2-3 **Experimental section**

S4-11 **Supplementary figures and images**

S12 **References**

Experimental section

Reagents. 2,3-Diaminonaphthalene (DAN), methylglyoxal (MGO), formaldehyde (FA), pyruvate (PYR), glyoxal (GO), benzaldehyde (BA), glyoxylic acid (GOA), glutaraldehyde (GA), *o*-phthalaldehyde (OPA), and 1-phenyl-1,2-propanedione (PPD), *o*-diaminobenzene (OPD), and dimethyl sulfoxide (DMSO) were purchased from Aladdin Chemical Industries, Ltd (Shanghai, China). NOC-7: 1-hydroxy-2-oxo-3-(N-methyl-3-aminopropyl)-3-methyl-1-triazene (a spontaneous nitric oxide releaser) was obtained from Sigma-Aldrich Chemical Industries, Ltd (Shanghai, China). WST-1 reagent was obtained from Roche Molecular Biochemicals (Mannheim, Germany). Environmental tissue specimen fixative was purchased from Kang Naixin Biomedical Technology Co., Ltd (Zhongshan, China). HeLa cells were purchased from Cell Resource Center of Shanghai Institutes for Biological Sciences, CAS. HSF cells were purchased from Dingguo Changsheng Biotechnology Co., Ltd (Beijing, China). Healthy type II diabetic KK-Ay mice were acquired from Hua fukang Biotechnology Co., Ltd (Beijing, China). The commercial mice MGO ELISA kit was purchased from Enzyme-linked Biotechnology Co., Ltd (Shanghai, China). Mice were housed in pairs under specific pathogen-free conditions, and were provided ad libitum access to food and water. All the mice were treated humanely throughout all experiments. All animal protocols were reviewed and approved by the Fuzhou University Institutional Animal Care and Use Committee. All experiments were performed in accordance with relevant guidelines and regulations. All reagents were of analytical grade and used as received.

Instruments. Fluorescence spectra were recorded on a F-4600 fluorescence spectrophotometer (Hitachi, Japan) equipped with a xenon lamp. UV-vis absorption spectra were obtained with a TU-19 UV-visible absorption spectrophotometer (Purkinje, Beijing) and microplate reader (Thermo scientific, USA). Mass-to-charge ratio (m/z) was determined by the Exactive Plus Orbitrap HPLC-MS system (Thermo Fisher Scientific, USA). ^{1}H and ^{13}C NMR were recorded by using AVANCE III 400 MHz spectrometer (Bruker, Switzerland). Cell and tissue imaging was captured through the Ts2R-FL (Nikon, Japan) and Multizoom AZ-C2+ (Nikon, Japan) laser fluorescence microscope system.

Preparation of MGO test strip. The filter paper was cut into 3.0*1.0 cm^2 test strips, immersed in 1 mM DAN ethanol solution for 15 min, and taken out and dried at room temperature for later use.

Cell culture. HeLa and HSF cells were cultured in 75 cm^2 tissue culture flasks with cell culture growth medium at 37 ^{o}C under a humidified atmosphere containing 5% CO_2. The cell culture medium for HeLa cells was EMEM supplemented with 10% FBS, 100 units/mL penicillin, 4 mM L-glutamine, and 100 μg/mL streptomycin. The cell culture medium for HSF cell was DMEM supplemented with 10% FBS, 100 units/mL penicillin and 100 μg/mL streptomycin. Cells were harvested after they reached 80% confluence and then resuspended in cell culture medium with the cell density of 5×10^4 cells/mL for further use.

Live and dead staining. A volume of 1 mL cell suspension (HeLa and HSF) was seeded into 24-well plate and culture the cells for 24 h. Afterward, the culture medium was removed and the DAN or MGO with concentration of 0, 20, 40, 60, 80, and 100 μM (diluted in cell culture medium) were added and being cultured another 24 h. Subsequently, live and dead cells on the well plate were visualized by using the Live/Dead assay kit according to manufacturer's protocol. The cells in each well were rinsed twice with PBS, and then added 400 μL of Live/Dead stock solution. After incubation at 37 ^{o}C for 15 min, the cells were observed by an inverted fluorescence microscope.

WST-1 assay. In addition, the cell viability was quantitatively analyzed by WST-1 assay. A volume of 100 μL cell suspension (HeLa and HSF) was seeded into 96-well plate and culture the cells for 24 h. Afterward, the culture medium was removed and the DAN or MGO with concentration of 0, 20, 40, 60, 80, and 100 μM (diluted in cell culture medium) were added and being cultured another 24 h. Subsequently, the cells in each well were rinsed twice with PBS, and then added 100 μL of cell culture medium and 10 μL of WST-1 reagent. After incubation at 37 ^{o}C for 3 h, the optical intensity in each well was measured at 450 nm using a microplate reader.

Supplementary figures and images

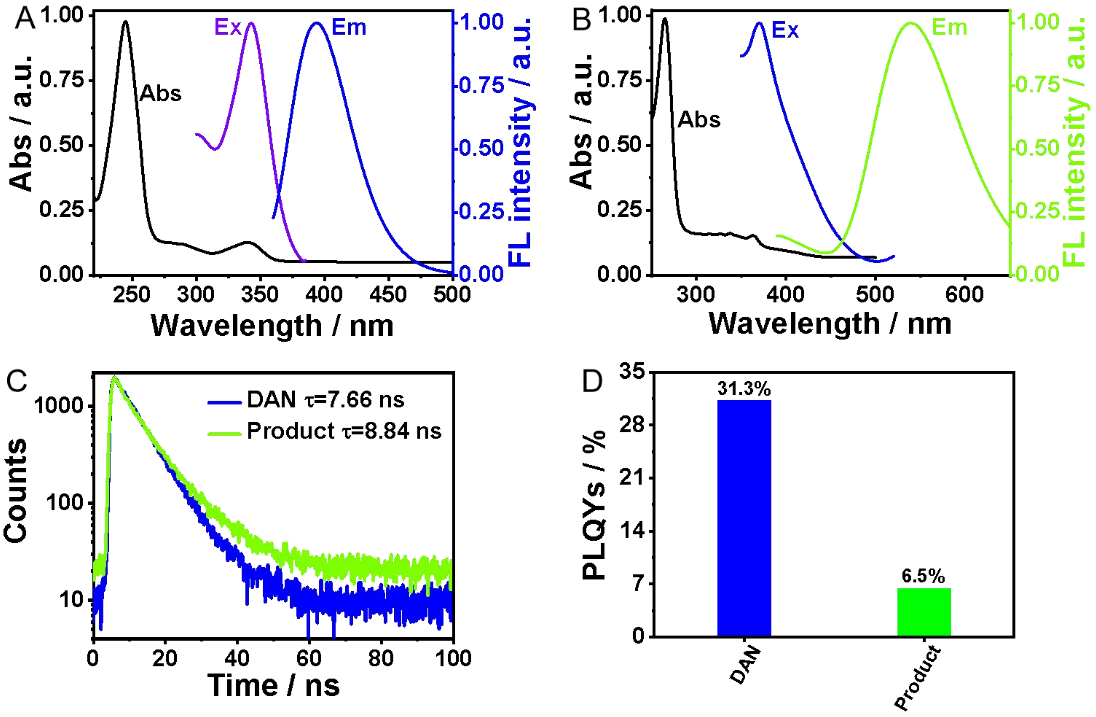

Figure S1. Fluorescence properties of DAN and product. (A) The optimal emission wavelength of DAN was located at 387 nm under 336 nm excitation. (B) The optimal emission wavelength of product was located at 544 nm under 365 nm excitation. (C) Time-resolved fluorescence decay curves. (D) The absolute photoluminescence quantum yield of DAN and product.

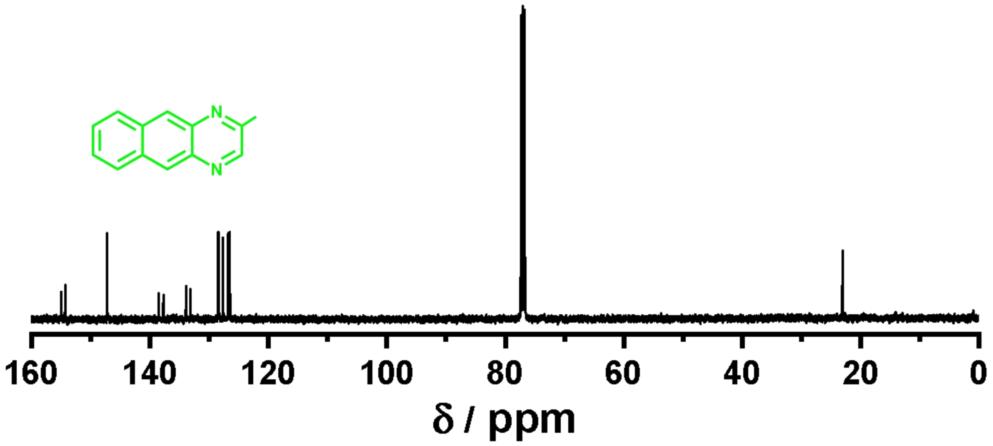

Figure S2. Partial ^{13}C NMR spectra of product ($CDCl_3$, 400 MHz).

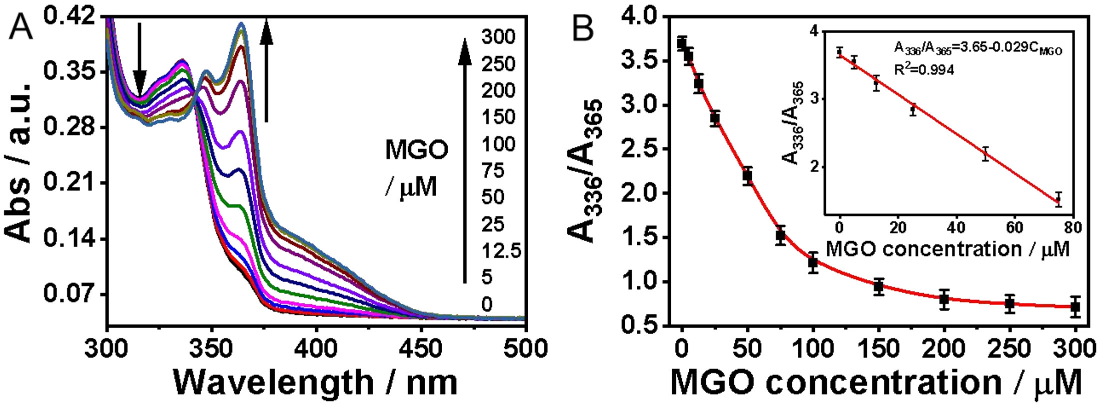

Figure S3. (A) UV-vis absorption spectra of DAN after adding different concentrations of MGO (0-300 μM) in PBS buffer (10 mM, pH 7.4, containing 0.05% DMSO) at 37 °C. (B) Plot of the ratio of A_{336}/A_{365} versus MGO concentrations

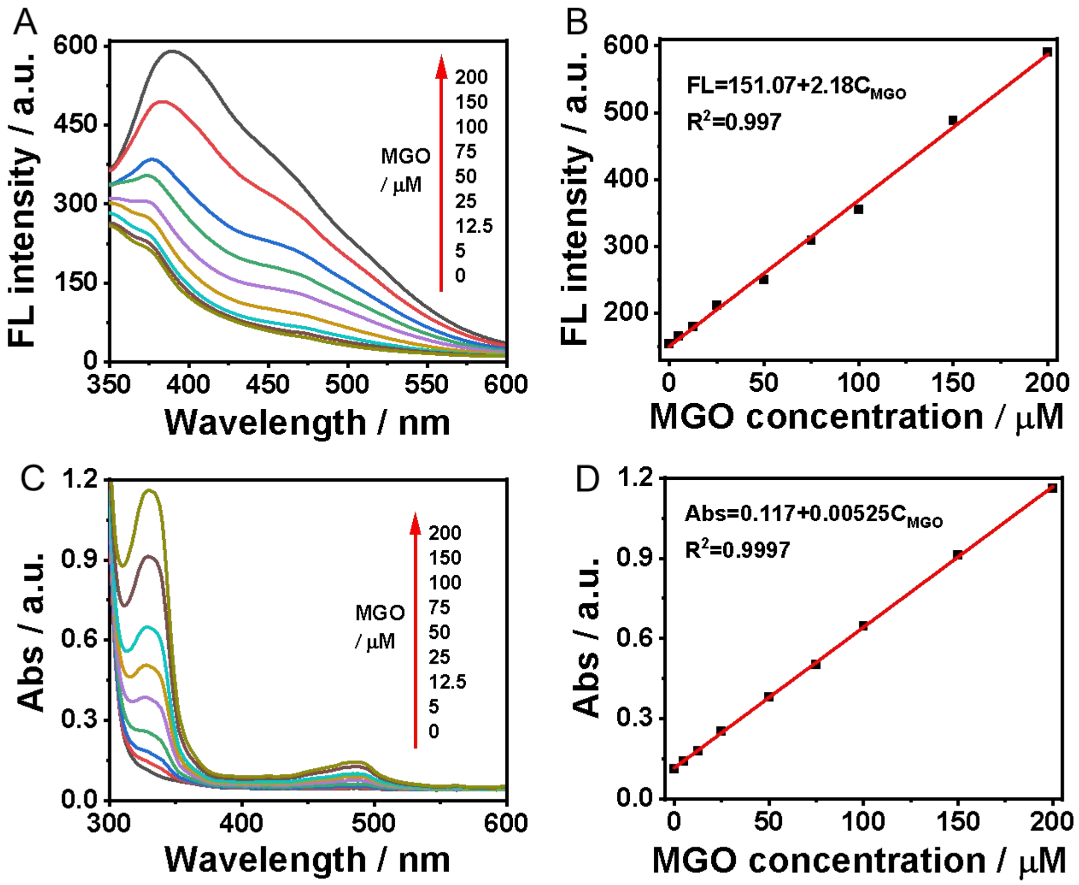

Figure S4. Traditional OPD-based method for the detection of MGO. Fluorescence spectrum (A) and the UV-vis absorption (C) of OPD responses to different concentrations of MGO (0–200 μM). Plot of fluorescence intensity (B) and absorption value (D) toward MGO concentrations.

Table S1. Comparison of the analytical performance of different methods for the detection of MGO.

Method	Linear range / μM	LOD / μM	Detection time / min	Reference
**HPLC**	0.2-1.0	0.0459	120	[1]
**Colorimetric**	---	0.25	30	[2]
**Fluorescence**	2-300	0.78	120	[3]
**Electrochemical**	3-30	0.24	3.3	[4]
**Fluorescence**	0-600	0.5	90	[5]
**Fluorescence**	0-75	0.326	60	**This work**

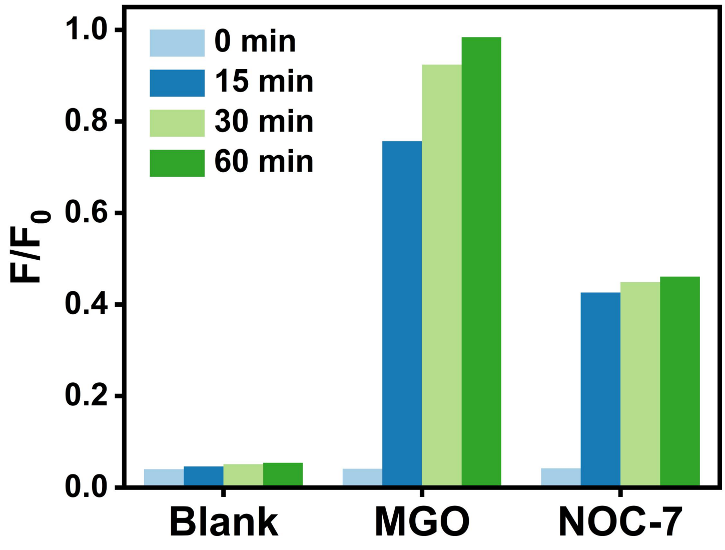

Figure S5. Normalized fluorescent intensity of adduct at 530 nm after DAN (20 μM) response to MGO and NOC-7. The reaction solutions contained MGO (50 μM) and NOC-7 (25 μM, releases two equivalents of NO), respectively. Blank: PBS buffer only; MGO: methylglyoxal; NOC-7: 1-hydroxy-2-oxo-3-(N-methyl-3-aminopropyl)-3-methyl-1-triazene (a spontaneous nitric oxide releaser). The nitric oxide releaser was fresh prepared before detection. All solutions were incubated at 37 °C in PBS buffer (10 mM, pH 7.4, containing 0.05% DMSO).

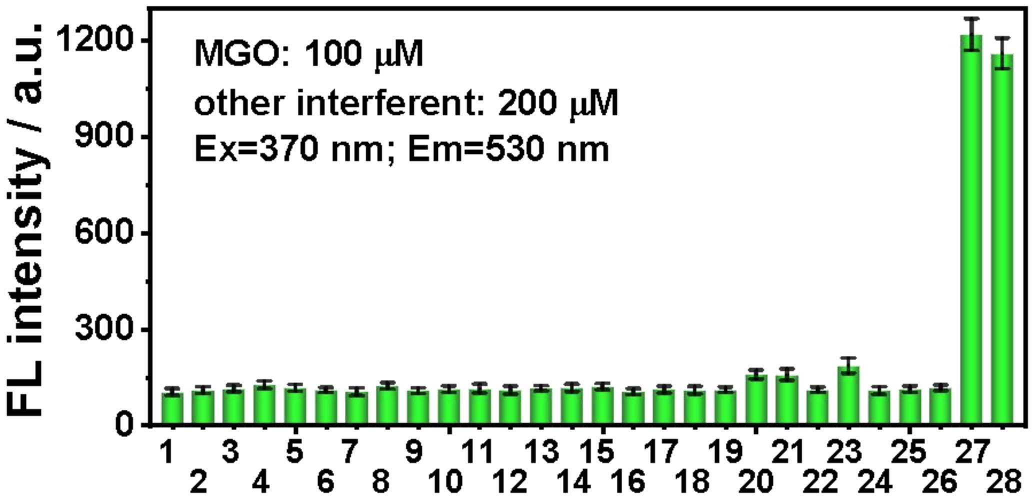

Figure S6. Responses of other common interferents (from 1 to 28, concentrations of interferents were 200 μM): (1) Na^{+}, (2) K^{+}, (3) Al^{3+}, (4) Pb^{2+}, (5) Ca^{2+}, (6) Cu^{2+}, (7) Fe^{3+}, (8) Mg^{2+}, (9) Zn^{2+}, (10) Cl^{-}, (11) Br^{-}, (12) I^{-}, (13) NO_3^{-}, (14) NO_2^{-}, (15) SO_3^{2-}, (16) SO_4^{2-}, (17) CO_3^{2-}, (18) PO_4^{3-}, (19) ascorbic acid, (20) glutathione, (21) L-cysteine, (22) HSA, (23) glucose, (24) ClO^{-}, (25) H_2O_2, (26) NO , (27) MGO, and (28) mixture, respectively.

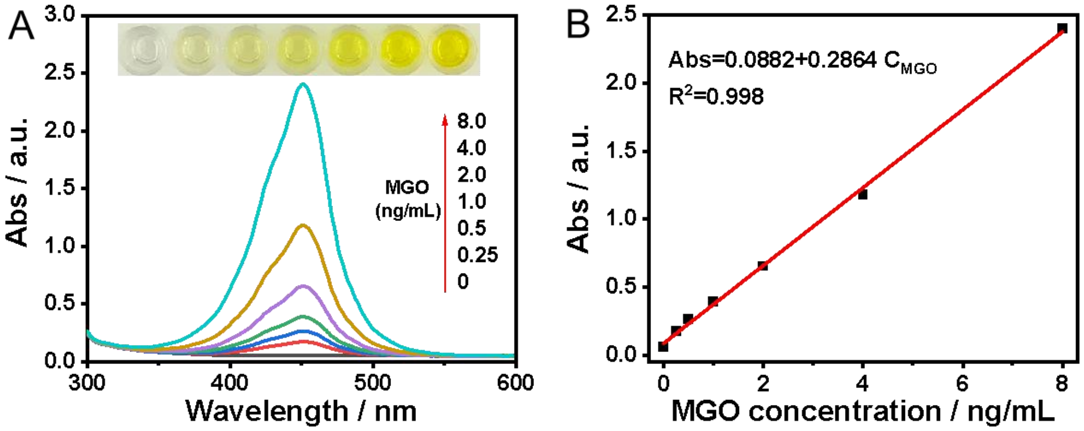

Figure S7. The commercial mice MGO ELISA kit for the detection of MGO. (A) The UV-vis absorption spectrum of TMB adding different concentrations of MGO (0–8.0 ng/mL). (B) Plot of the absorption of TMB against MGO concentration. The maximum absorption wavelength of TMB^{2+} is located at 450 nm.

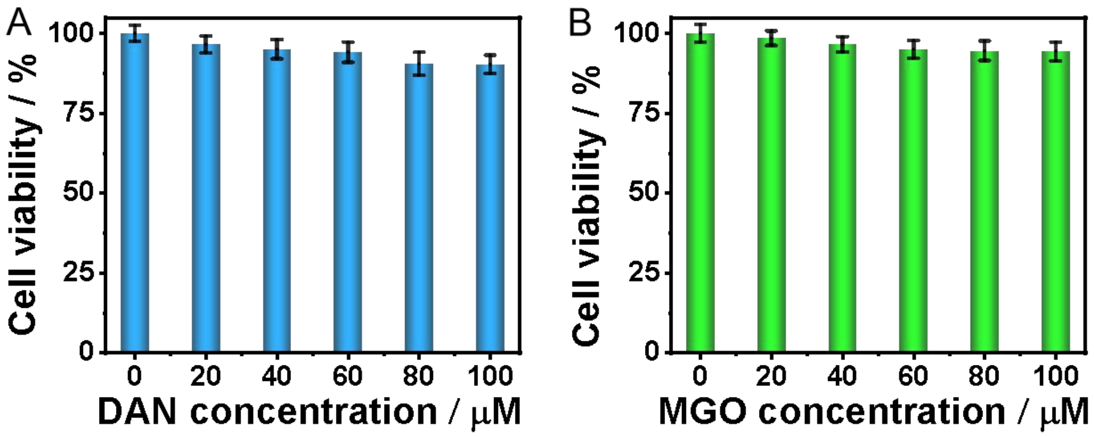

Figure S8. Cell viability of HSF cells stained with DAN (A) and MGO (B) at various concentrations (0, 20, 40, 60, 80, and 100 μM) for 24 h at 37 °C.

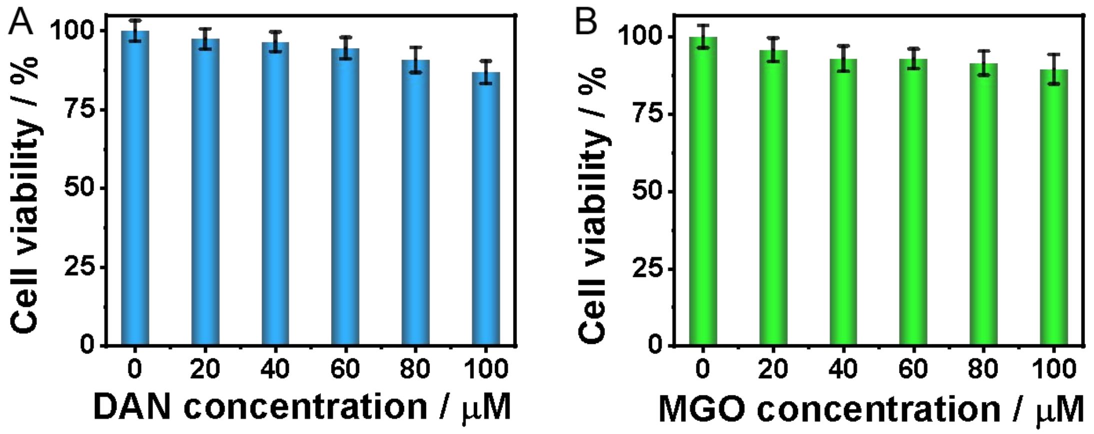

Figure S9. Cell viability of HeLa cells stained with DAN (A) and MGO (B) at various concentrations (0, 20, 40, 60, 80, and 100 μM) for 24 h at 37 °C.

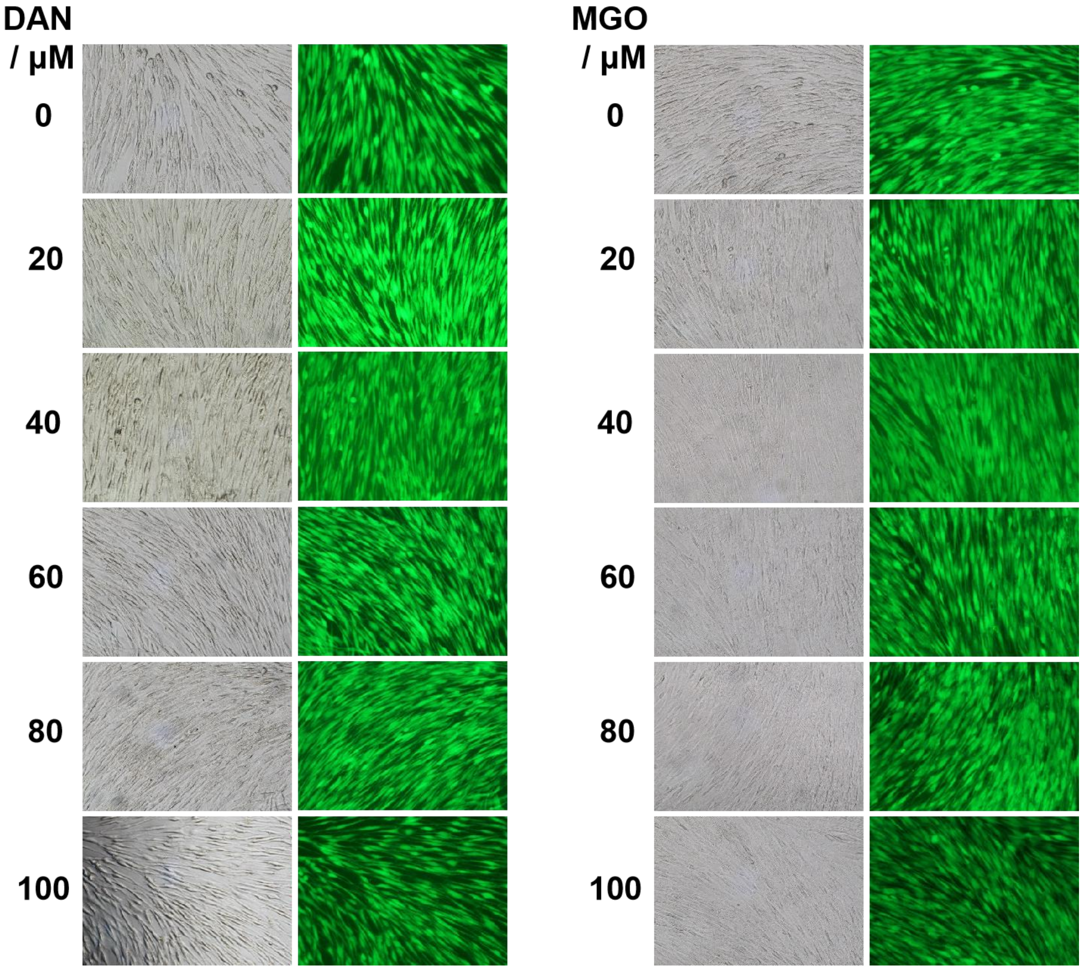

Figure S10. Live and dead staining of HSF cells towards different concentrations of DAN and MGO.

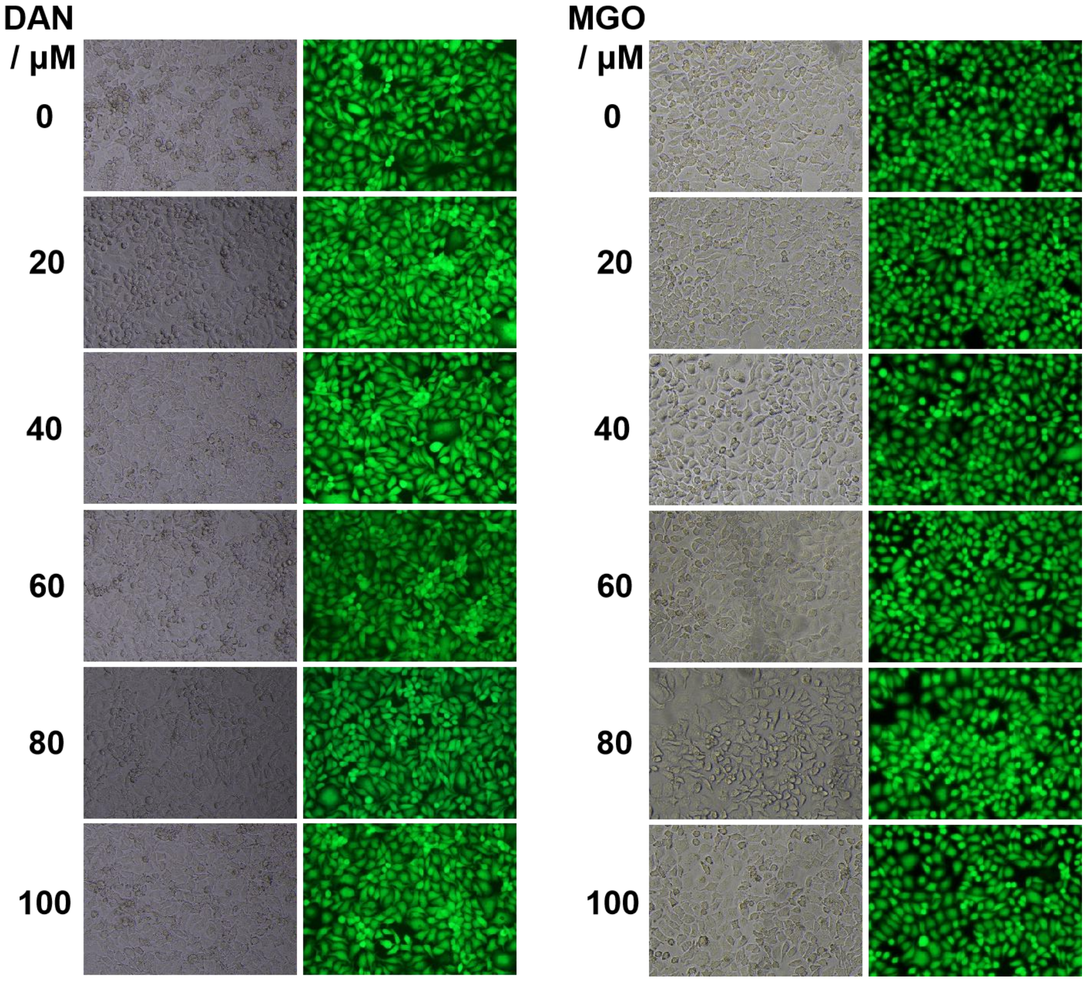

Figure S11. Live and dead staining of HeLa cells towards different concentrations of DAN and MGO.

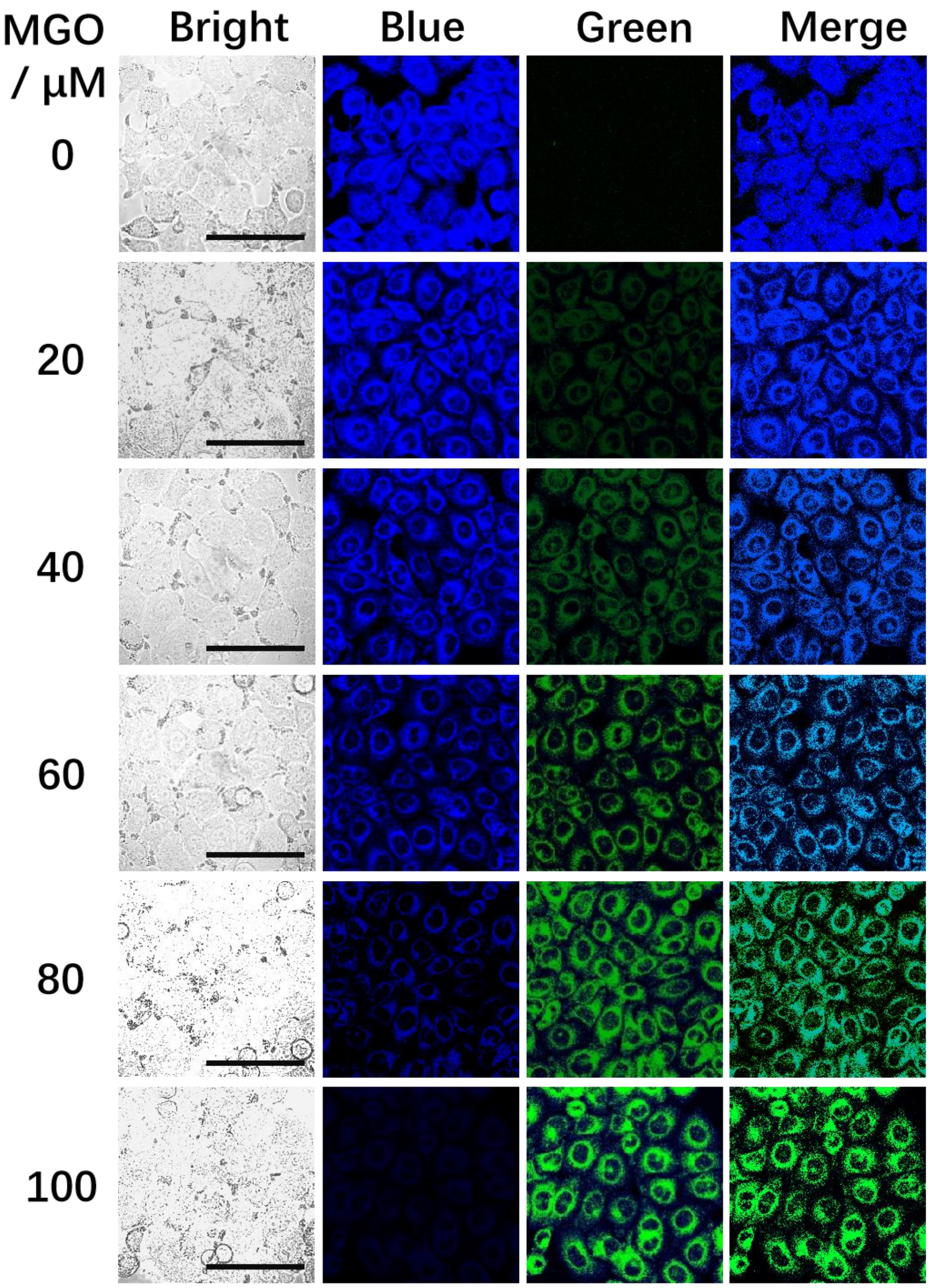

Figure S12. Fluorescence confocal ratiometric imaging of exogenous MGO in HeLa cells. Scale bar: 50 μm.

References

[1] Nemet, I.; Varga-Defterdarovic, L.; Turk, Z. *Clin. Biochem.* **2004**, *37*, 875-881.

[2] Wang, S. T.; Lin, Y.; Spicer, C. D.; Stevens, M. M. *Chem. Commun.* **2015**, *51*, 11026-11029.

[3] Zhang, W.; Zhang, F.; Wang, Y. L.; Song, B.; Zhang, R.; Yuan, J. *Inorg. Chem.* **2017**, *56*, 1309-1318.

[4] Ramachandra Bhat, L.; Vedantham, S.; Krishnan, U. M.; Rayappan, J. B. B. *Biosens. Bioelectron.* **2018**, *103*, 143-150.

[5] Wang, H.; Xu, Y.; Rao, L.; Yang, C.; Yuan, H.; Gao, T.; Chen, X.; Sun, H.; Xian, M.; Liu, C.; Liu, C. *Anal. Chem.* **2019**, *91*, 5646-5653.
